# Cardiac sarcoidosis presenting as recurrent ventricular tachycardia: a case report

**DOI:** 10.1186/1757-1626-2-9353

**Published:** 2009-12-18

**Authors:** Mir Yasir, Imran Masood, Aiffa Aiman, Sheikh Afaq, Ashok Bakaya

**Affiliations:** 1 Department of Surgery, ASCOMS & Hospitals, Sidhra, Jammu, J&K, 180017, India; 2 Department of Medicine, ASCOMS & Hospitals, Sidhra, Jammu, J&K, 180017, India; 3 Department of Pathology, GMC, Bakshinagar, Jammu, J&K, 180017, India

## Abstract

**Introduction:**

Sarcoidosis is a multi-systemic disorder involving various organ systems. Though cardiac involvement is uncommon it can present as life threatening arrhythmias and sudden death.

**Case presentation:**

Here we present 27 years old married female with a series of arrhythmias with no initially obvious aetiology. On further evaluation she was diagnosed as having cardiac sarcoidosis.

**Conclusion:**

Cardiac sarcoidosis is an important cause of death in patients with systemic sarcoidosis. It is therefore necessary to have high index of suspicion when symptoms are present rather than ignoring them.

## Introduction

Sarcoidosis is a multi-systemic, granulomatous disease with occasional cardiac involvement [1]. Cardiac sarcoidosis may cause various symptoms including congestive cardiac failure, arrhythmias, conduction disturbance and sudden death depending on the extent and site of cardiac involvement [2,3]. We describe a patient of cardiac sarcoidosis presenting with recurrent ventricular tachycardia.

## Case presentation

27 years old married Indian female with Indoaryan ethinicity presented to the hospital with a history of sudden onset palpitation, sweating with cold hands and feet, since the last 3 months. These symptoms were intermittent and usually used to last for 1-5 minutes. There was no history of syncope, chest pain, breathlessness, hemoptysis, fever, history suggestive of rheumatic heart disease or any substance abuse. 1 year back patient had fever which lasted for 2 weeks along with enlarged preauricular lymph node.

FNAC of the node had revealed it to be a non-caseating granulomatous pathology. Patient was put on anti-tubercular therapy by family physician that she continued for 9 months. There is also history of anterior uveitis 6 months back and 4 months back she had infra-nuclear type of facial palsy. She had complete recovery from these symptoms. She was put on proton pump inhibitors since last 3 months by her treating physician attributing her complaints of palpitation and uneasiness to some epigastric discomfort.

On examination patient was conscious, oriented, had mild pallor but no icterus, cyanosis, edema or clubbing. She had a small non-tender lymph node palpable, in her left submandibular region. Her blood pressure was 100/70 mmHg with no postural drop, Pulse; 80/mt, regular. She was afebrile and had no features of respiratory distress.

**Figure 1:**
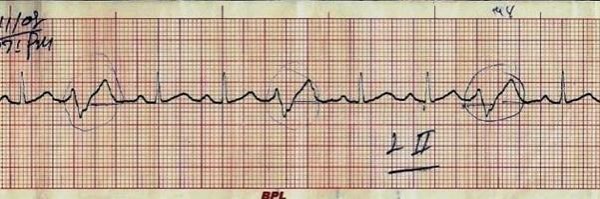
**ECG of the patient showing ventricular ectopics (Trigeminy) during the episode of palpitation**.

**Figure 2:**
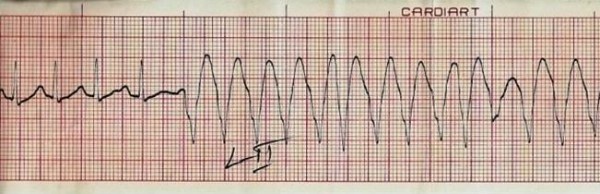
**ECG of the same patient revealing ventricular tachycardia preceded by normal sinus rhythm**.

Investigations revealed Hb-9 g/dl, W.B.C count- 8000/μL, Platelet count- 2 lakh/μL, E.S.R- 30 mm, Peripheral blood film- mild hypochromic picture, Blood sugar- 60 mg/dl, Urea-21 mg/dl, creatinine- 1 g/dl, Albumin- 3.5 g/dL, SGOT- 137 U/L, SGPT- 101 U/L, Alkaline Phosphatase- 111 U/L, Serum Calcium- 1.0 mmol/l, Serum Sodium- 137 meq/l, Serum Potassium- 3.7 mEq/l. Serum amylase- 72 U/L. Her X-Ray chest showed bilateral hilar prominence. Angiotensin converting enzyme levels were 209.7 (Normal = 65 to 114.4). An electrocardiogram showed ventricular ectopics (Trigeminy) during the episode of palpitation (Figure 1). She was put on beta-blocker and a holter cardiac study was done. Patient was managed in cardiac care unit for continuous ECG monitoring. On continuous cardiac monitoring it was found that she was having recurrent sustained ventricular tachycardias completely coinciding with her feeling of palpitation and sweating (Figure 2). Each Ventricular tacvhycardia lasted for 30 sec to 2 minutes. Her blood pressure remained stable during the arrhythmias. After a loading dose of continuous infusion of amiodarone, frequency of stable Ventricular tacvhycardias decreased but persisted and a she was then chemically cardioverted with continuous infusion both amiodarone and lidocaine. Echocardiography was done which showed only trivial Mitral Regurgitation, with no evidence of Congestive heart failure with preserved ejection fraction.

**Figure 3:**
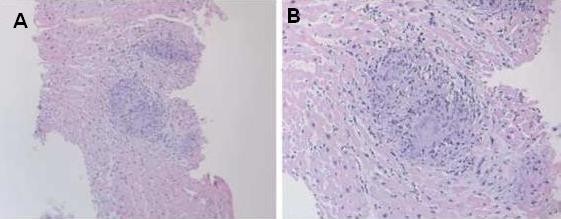
**Histology (haematoxylin and eosin stain) showing non-caseating granuloma with multinucleate giant cells at (A) low (×100) and (B) high (×200) magnification**.

CECT chest showed significant anterior mediastinal and bilateral hilar lymphadenopathy with FNAC of hilar nodes showing features of non-caseating granuloma. Endomyocardial biopsy was performed at the interventricular septum. Cardiac biopsy showed non-caseating granulomata, highly suggestive of a diagnosis of cardiac sarcoidosis (Figure 3). A diagnosis of cardiac sarcoid was made on the basis of these CECT findings, histology, and the clinical picture.

Patient was simultaneously put on prednisolone 60 mg/day and shifted to oral amiodarone 200 mg tid. Due to poor financial status of the patient, implantation of intracardiac defibrillator was not possible in our case. Patient was discharged in a good clinical condition after 7 days of hospital stay.

## Discussion

Sarcoidosis should always be considered in differential diagnosis of ventricular tachycardia of unidentified cause. Cases of sudden death in patients of cardiac sarcoidosis have been reported. Arrhythmia control in cardiac sarcoidosis is difficult, and all modern treatment including high dose of steroids, anti-arrhythmic, implantable intracardiac defibrillators and catheter ablation are needed to suppress the arrhythmia [4]. Prospective trial data do not exist, but spontaneous VT, severe LV dysfunction, and severe intraventricular conduction disturbance warrant ICD and/or pacemaker therapy as appropriate [5]. Cardiac sarcoidosis is an important cause of death in patients with systemic sarcoidosis [6]. It is therefore necessary to have high index of suspicion when symptoms are present rather than ignoring them. The prognosis of myocardial sarcoidosis is poor and depends on arrhythmias and conduction disorders [7,8].

## Consent

Written informed consent was obtained from the patient for publication of this case report and accompanying images. A copy of written consent is available for review by Editor-in-Chief of this journal.

## Competing interests

The authors declare that they have no competing interests.

## Authors' contributions

MY gave design, concept and helped in writing the manuscript. IM interacted with the patient and his family and helped in analysis and interpretation of data. AA helped in performing all the necessary investigations. SA was involved in revising the patient data and management of the patient. AB guided in interpretation of case, diagnosis, guidance and approval of the final manuscript.
